# Fractal solar panels: Optimizing aesthetic and electrical performances

**DOI:** 10.1371/journal.pone.0229945

**Published:** 2020-03-10

**Authors:** Ellis T. Roe, Alexander J. Bies, Rick D. Montgomery, William J. Watterson, Blake Parris, Cooper R. Boydston, Margaret E. Sereno, Richard P. Taylor

**Affiliations:** 1 Department of Physics, University of Oregon, Eugene, Oregon, United States of America; 2 Department of Psychology, Gonzaga University, Spokane, Washington, United States of America; 3 Department of Psychology, University of Oregon, Eugene, Oregon, United States of America; Nanyang Technological University, SINGAPORE

## Abstract

Solar energy technologies have been plagued by their limited visual appeal. Because the electrical power generated by solar panels increases with their surface area and therefore their occupancy of the observer’s visual field, aesthetics will play an increasingly critical role in their future success in urban environments. Inspired by previous psychology research highlighting the aesthetic qualities of fractal patterns, we investigated panel designs featuring fractal electrodes. We conducted behavioral studies which compared observers’ preferences for fractal and conventional bus-bar electrode patterns, along with computer simulations which compared their electrical performances. This led us to develop a hybrid electrode pattern which best combines the fractal and bus-bar designs. Here we show that the new hybrid electrode matches the electrical performance of bus-bars in terms of light transmission and minimizing electrical power losses, while benefiting from the superior aesthetics of fractal patterns. This innovative integration of psychology and engineering studies provides a framework for developing novel electrode patterns with increased implementation and acceptance.

## Introduction

The cost of solar panels has more than halved since 2010 [1], triggering a rise in their popularity [2]. For example, the California Energy Commission recently voted to make rooftop solar panels a mandatory addition to all new single-family homes commencing in 2020 [3]. However, their large surface areas will significantly impact our daily visual experiences. The psychology of aesthetics will therefore play an increasingly critical role in solar panels’ success, especially as aesthetics has been shown to be a major concern when considering solar technologies [4]. Recent ‘blended’ designs seek to neutralize the poor aesthetics of traditional panels by covering them with camouflaging louvers which visually match the panels to their surroundings [5]. Rather than neutralizing their visual impact, we propose panels that actively enhance the environmental aesthetics.

Solar technology has come a long way since the first practical silicon solar cell was developed at Bell Labs in 1954. Today’s panels typically feature an array of 10x10cm solar cells which employ electrodes on their top surface to transmit electricity generated by the underlying photodiode to an external circuit [6–7]. The traditional ‘bus-bar’ electrode design features horizontal ‘fingers’ (each 50–200μm wide) intersected by several ‘bars’ (1-2mm wide). The number of fingers and bars vary between designs with the goal of optimizing their optical and electrical efficiencies. Some designs even position the electrode at the photodiode’s back leaving it fully exposed to light [8].

Finding the optimal electrode pattern is a challenging problem on its own. Increases in efficiency produced by reducing the light blockage by electrodes competes with minimizing the power losses experienced by the generated electricity as it flows out of the cell [9]. The solar industry adopted the bus-bar because its so-called ‘multi-layered’ approach of intersecting bars and fingers out-performs simpler, single-layer approaches in its capacity to effectively balance the competing factors [10]. The bus-bar’s efficiency can be calculated analytically [11] and today this efficiency is then weighed against the quantity of silver required to construct the electrode patterns as growing silver costs are of concern [12].

To explore whether aesthetics can be successfully incorporated into this optimization process, we turned to biophilia [13–16]. This well-established movement employs natural patterns in architectural and engineering applications [13–16]. Pioneering studies demonstrated that exposure to natural scenery can have dramatic, positive consequences for the observer, even accelerating patients’ recovery from major surgery [17–18]. A range of visual factors could contribute to biophilia. Given that many natural objects feature the repeating patterns of fractals [19], a ‘fractal fluency’ model proposed that the visual system has adapted to these fractals through exposure, allowing us to efficiently process the visual complexity generated by their pattern repetition [20–22]. Studies have quantified a range of positive responses to viewing both natural fractals and their computer-generated imitations, including aesthetic experiences [23–35] and physiological stress reduction [36]. The World Health Organization suggests stress is becoming a major health epidemic, with stress-related illnesses costing countries billions of dollars annually [37]. Given their prevalence in nature, and the positive effects they induce in the observer, incorporation of fractals into the built environment represent an invaluable opportunity for the biophilic movement. More specifically, fractal solar panels could realize the health benefits of biophilia while simultaneously promoting sustainable energy.

In addition to maximizing aesthetics, it is paramount to preserve efficient energy transfer using the solar electrodes. Nature’s fractals have previously served as bio-inspiration to enhance performances in diverse applications from wind barriers [38] to capacitors [39]. Most relevant for solar panels, fractal electrodes have been shown to out-perform Euclidean electrodes in simulations of retinal implants which use photodiodes to restore human vision [40–41]. This raises the possibility that fractal electrodes in solar panel photodiodes might surpass bus-bars aesthetically and electrically.

Here, we tested the hypothesis that incorporating fractals into solar panel electrode designs will enhance their aesthetic and electrical properties. To accomplish this task, the interdisciplinary team of psychologists and physicists adopted an iterative process of refining the designs based on their two distinct functions. Firstly, we performed a series of experimental aesthetics studies which revealed that observers preferred fractal over conventional bus-bar patterns and also identified the fractal characteristics that maximized this preference. We then performed simulations using Modified Nodal Analysis [42] to quantify the patterns’ electrical performances. These highlighted the need for a novel hybrid design that combines the electrical efficiency of the bus-bar with the superior aesthetics of the fractal pattern. Subsequent aesthetic and electrical studies confirmed these performances. Although our specific study gravitated to the optimal condition quickly, our approach could employ many iterations for other studies. Our hope is that this novel iterative exchange between disciplines will become a standard approach for biophilic studies and, more generally, science-informed design.

## Methods

### Methods for the behavioral studies

#### Participants

The IRB at the University of Oregon approved the study (Approval # 090702010.006). Written consent was obtained from participants. Participants were students at the University of Oregon (UO) who chose to complete a survey for course credit. For Study 1, the 36 participants (20 female, 16 male) ranged in age from 18 to 67 (median age 19) and consisted of 25 Caucasian, 5 Asian, 1 African American, 1 other, and 1 individual who declined to answer. For Study 2, the 34 participants (18 female, 16 male) ranged in age from 18 to 27 (median age 19) and consisted of 26 Caucasian, 5 Asian, 1 African American and 1 other. For Study 3, the 299 participants (201 female, 91 male, 1 other, 6 did not respond to the question) ranged in age from 18 to 85 (median age 19.46) and consisted of 211 Caucasian, 36 Asian, 26 Hispanic or Latino, 8 African American, 4 Native Hawaiian or other Pacific Islander, 4 American Native, 1 African and 10 others.

#### Procedure

For each survey, participants signed in to our website for research with human subjects from locations of their choice and opted to complete the survey. On the survey’s first page, participants were shown our informed consent documentation, including a note that they would not be penalized for failing to complete the survey or for skipping items. After consent, they were asked several demographic questions (age, gender and ethnicity/race), several unrelated scales on topics including clinical and personality psychology (the content of these varied randomly across participants and is not presented here because they were items from other research groups’ projects and are not relevant to the current study) and questions related to preferences of visual stimuli. For each study, participants were asked to select the image on each page that they found to be “the most visually appealing” by clicking a button presented to the left of the chosen image. On each page, images were aligned vertically, such that the participant could scroll through to view each image. Survey items were repeated with images in reverse or random orders as a check for insufficient effort responding. Participants could not return to previous pages in the survey.

**Fig 1:**
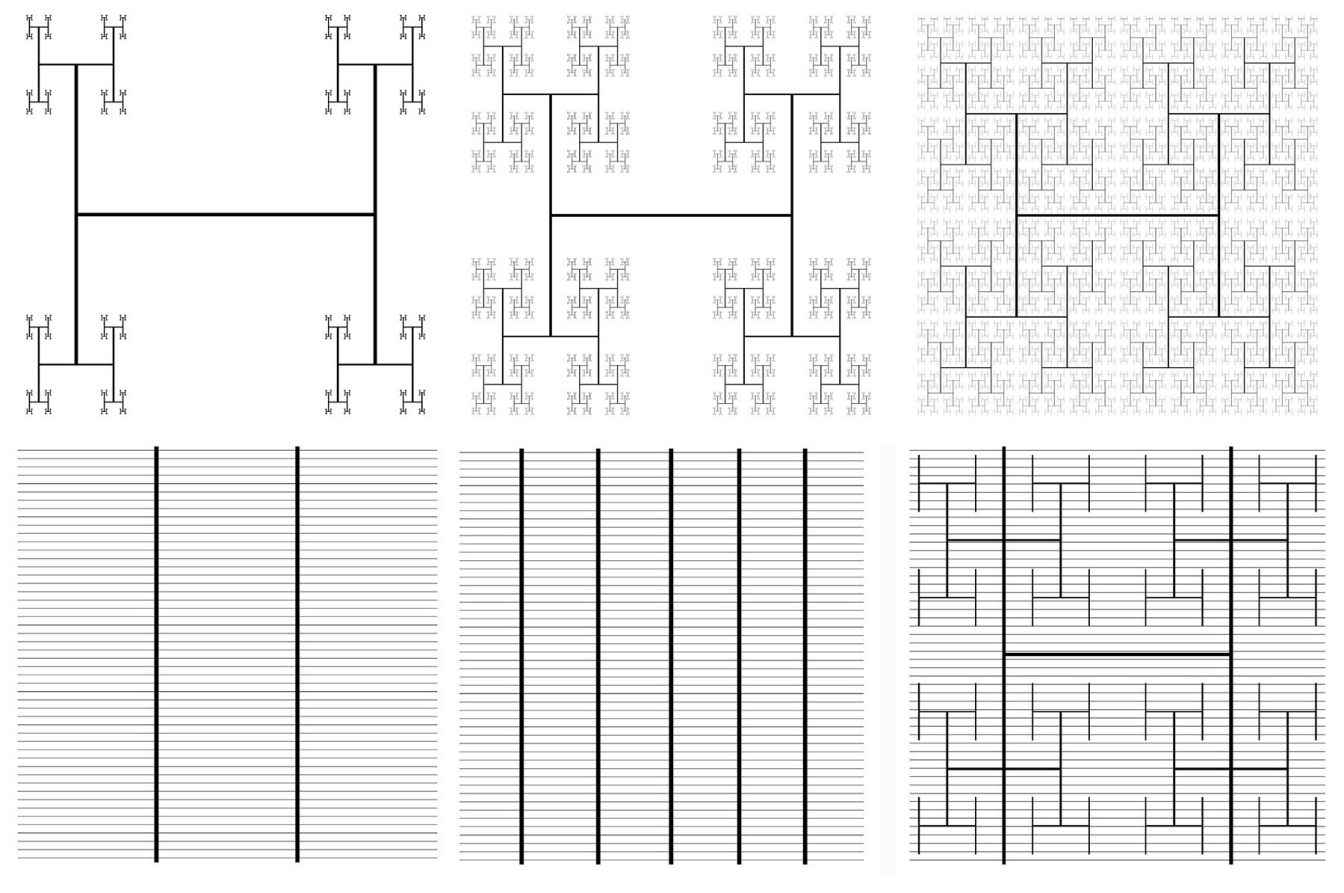
Fractal H-trees and bus bar patterns. Top row: H-trees with *D* = 1 (left), 1.5 (middle), and 2 (right) and *m* = 6. Bottom row: Bus-bar with 2 vertical bars (left), bus-bar with 5 vertical bars (middle) and a hybrid pattern integrating a *D* = 2, *m* = 3 H-tree with a 2 bus-bar (right). Each pattern in the bottom row features 50 horizontal finger lines.

The stimuli for the study were constructed as follows. Fractals consist of a ‘seed’ pattern that repeats at different magnification levels with the reduction in pattern size between the levels set by a power law. H patterns were chosen as the seed for our fractals (see Discussion Section) and these patterns scale according to *N = L^−D^*, where *N* is a constant related to the chosen seed pattern. The visual impact of *N* is that it reflects the number of smaller H patterns added to each larger H pattern, which is *N* = 4 (Fig 1). *L* is the length scaling factor (i.e. the ratio of the pattern lengths for subsequent levels) Through the power law, the fractal dimension, *D*, therefore quantifies the rate at which the H shrinks between magnification levels. Fig 1 (top row) demonstrates the visual impact of this process: Smaller *D* patterns correspond to shrinking the patterns at a faster rate between levels. This procedure generates fractal patterns embedded in a 2-dimensional plane with *D* values in the range 1 < *D* < 2. We note that it is also possible to generate fractal patterns that extend into 3 dimensions with *D* values in the range 2 < *D* < 3. However, we did not consider these patterns because they deviate from the flat panel designs utilized by the solar industry (based on, for example, manufacturing cost considerations). We set the number of levels of the repetition process at *m* = 6 for the H-trees because for higher *m* the finest levels will not be visible either on the monitor of the behavioral studies (Fig 1) or when incorporated into solar panels (see General Discussion). In each case, the size of the coarse-scale H was selected such that H-trees with different *D* values are all contained within the same perimeter. This ensures that they all occupy the same solar cell area when incorporated into a panel.

We conducted three studies. These were performed over a series of academic terms with their choice of stimuli informed by the previous results. For Study 1, 11 H-tree images were presented to test preference across *D* (see Fig 1 for examples). For Study 2, a 6-item survey was used to test preference for H-tree images vs. a bus bar. On each page, an H-tree with *D* = 1, 1.5 or 2 was presented with a bus-bar (featuring 2 bars). For each *D* value, the H-tree was presented once above and once below the bus-bar. For Study 3, the 4 images (2 bus bar patterns, hybrid pattern and blank image) were presented sequentially on 2 pages. Image order was randomized on both pages for each participant.

#### Data analysis

In each study, data was discarded for participants who failed to complete the task or consistently selected images on the basis of their location in the stimulus array (e.g., the first option on every page). Two participants’ data were discarded from Study 1, while 89 participants’ data was discarded from Study 3. All other participants’ responses were retained. These participants’ responses were counted as frequencies in each analysis. Participants in Study 1 were categorized as preferring a particular level of *D* according to their response on the 1^st^ item. Participants in Study 2 were categorized as preferring H-tree or bus-bar at each level of *D* (1.0, 1.5, and 2.0). Participants in Study 3 were categorized as preferring the hybrid pattern, 2-bar bus-bar, 5-bar bus-bar, or blank image.

### Methods for the electrical simulations

**Fig 2:**
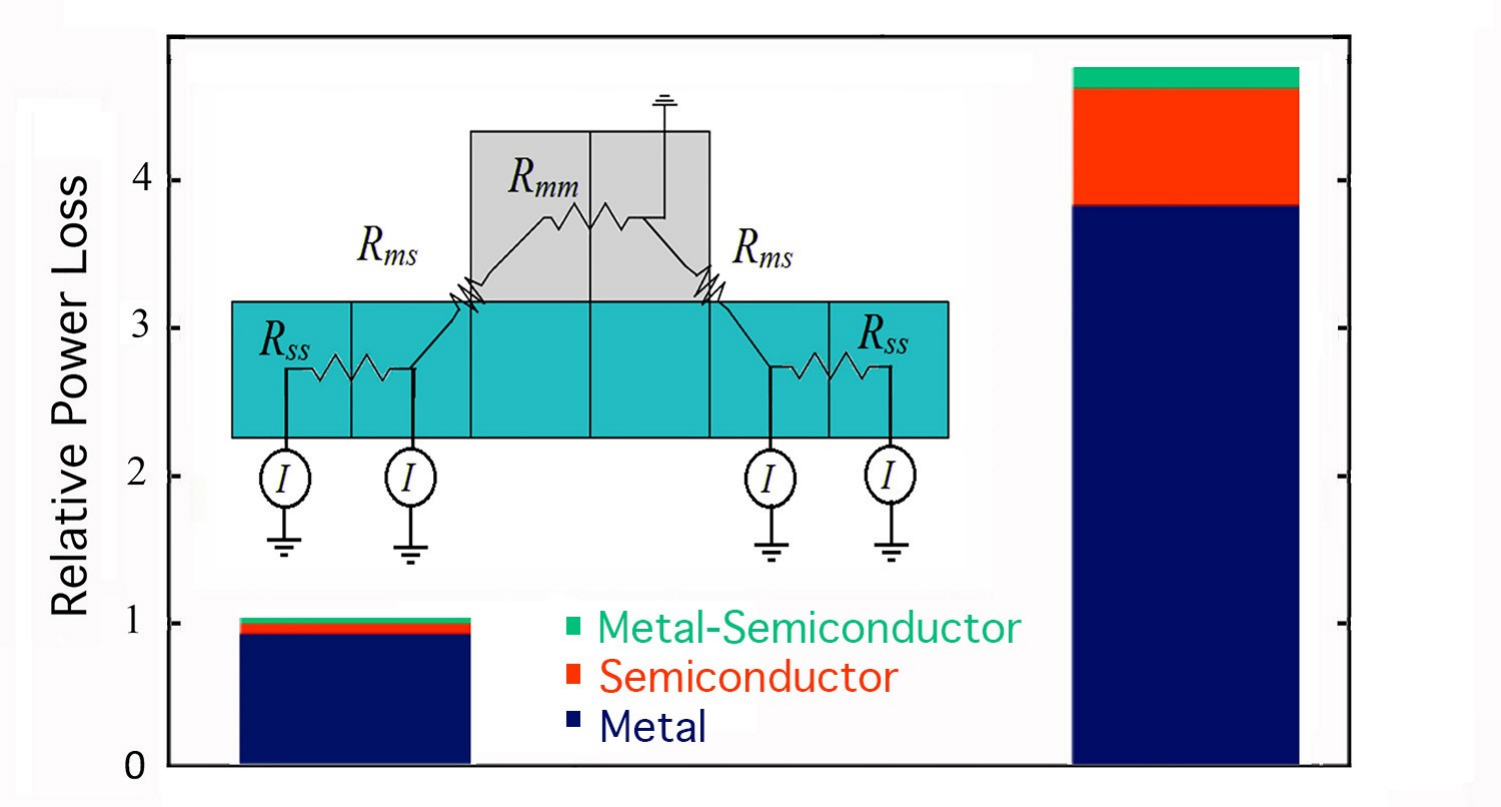
Power loss graph with semiconductor cross-section. Bar graphs of the power losses for cells featuring a bus-bar (left) and a H-trees (right), each covering 13.9% of the emitter’s surface area. The power losses are normalized such that the bus bar’s total power loss is unity. The power losses originate in the semiconductor (red), metal (blue) and the metal-semiconductor junction (green). The bus-bar featured 50 finger lines (width *W*_*f*_ = 100μm and gap *G*_*f*_ = 800μm) and 2 vertical bars (width *W*_*b*_ = 1mm and gap *G*_*b*_ = 3.3cm). The H-tree was quantified by *D* = 2 and *m* = 6, with an electrode width *W*_*h*_ spanning from 500μm for the coarsest H to ~100 μm for the finest H. Inset: Schematic showing a vertical cross-section of 6 semiconductor (light blue) and 2 metal (grey) pixels connected by resistive elements. Because the model operated in short circuit (i.e. with no load resistance), the pixel at which the electrode connected to the external circuit was grounded.

To simulate each 10 by 10cm solar cell, the emitter layer was modeled as a 1000 by 1000 array of 100μm-wide emitter pixels. For simplicity, we compared the electrical performance of the various electrode patterns when operated at short circuit (see below). Accordingly, each exposed emitter pixel (i.e. not located under the electrode) was modeled as a constant current source of *I* = 5 x 10^-6^A and excluded any diode behavior [40]. This current was then pictured as flowing up through the electrode via a network of nodes connected by resistive elements (Fig 2, inset). Using Modified Nodal Analysis [42], the electrical efficiency of the electrode patterns could then be compared by summing up the ohmic power losses in these elements.

Calculations of the semiconductor-semiconductor (*R*_*ss*_) connections were based on a sheet resistance of 55 Ω/□, while the metal-metal (*R*_*mm*_) connections each had a resistance of 5 x10^-4^Ω. The semiconductor-metal (*R*_*ms*_) resistance was determined using a ‘current crowding’ model [43] in which the current diffusing from the semiconductor to the electrode decays exponentially with distance *x* from the electrode as $I(x)=IExp(-\frac{x}{L_{T}})$, where the decay rate is set by the ‘transfer length’ *L*_*T*_. The junction resistance was calculated to be 0.74Ω using $R_{ms}=\frac{R_{ss}L_{T}}{H}$, where *H* is the emitter pixel width, *L*_*T*_ = $\sqrt{\frac{\rho_{c}}{R_{ss}}}$ and the contact resistivity *ρ*_*c*_ is 1 x 10^−6^ Ω cm^2^ [44]. By inputting the network of resistances and current sources into a Modified Nodal Analysis algorithm, the computation transforms the 1000 by 1000 pixels into a system of approximately 10^6^ equations with 10^6^ unknowns derivable using Kirchhoff’s circuit laws. The output is a voltage value for each node. Finally, we calculated the total power loss by summing $\frac{{\Delta V}^{2}}{R}$ for each node connection and then grouped these losses according to type: metal-metal, semiconductor-semiconductor, and junction.

Our model excluded electron-hole re-combination processes. Because the diffusion lengths (typically 1mm - 1cm) in monocrystalline silicon are significantly larger than the emitter layer thickness, recombination in the emitter is generally insignificant [45]. Recombination at the metal-semiconductor interface may be more significant, even with differential doping under the electrodes [45]. However, the feature sizes and covering area are similar for the various electrode designs and, as such, we expect the degree of recombination at their metal-semiconductor interfaces will not differ significantly. Additionally, these recombination rates are expected to be minimized at short circuit [46]. Ignoring this recombination will not therefore impact comparisons of power losses.

Although we examined the power losses in short circuit operation, any geometric limitations will persist when a load resistance is introduced into the circuit. By choosing short circuit, we compared power losses in the most pessimistic operating condition. When a load resistance is introduced, each emitter pixel is then modelled using a diode in addition to the current sources used in the model presented here. This decreases the current passing through the cell and therefore the total internal ohmic power loss of the solar cell.

Given that the current study simply compares the relative power losses of the H-tree, bus-bar and hybrid electrodes, the short circuit condition is sufficient to demonstrate their relative performances. If absolute power losses are of interest in future studies, we point out the computational challenges associated with calculating power losses for fractal electrodes operating away from open circuit. Simulations rely heavily on the relatively small numbers of elements in the bus bar design when solving the partial differential equations required when diodes are included in the model [46]. In contrast, the intricate structure of the fractal design requires exponentially larger computing power as multiple size-scales are introduced into the electrode design.

## Results and discussion

### Aesthetics of electrode patterns

Conventional solar panels featuring Euclidean electrode designs elicit negative aesthetic responses [43]. We therefore took inspiration from nature’s fractal geometry to develop a new electrode pattern, given an extensive literature supporting fractal patterns’ efficacy in engineering applications [47], health benefits [36], and aesthetic appeal [23–35]. Previous aesthetics studies have investigated a variety of ‘exact’ fractals which repeat patterns exactly at different scales [23]. For the current investigation, we generated a fractal previously unstudied in the aesthetics literature, the H-tree. This choice was based on ease of manufacture, given that the H-tree is closer in design to bus-bars than the previously researched patterns. For example, it is composed of perpendicular intersecting lines and is symmetric (patterns with sloping lines and asymmetries, such as the Koch snowflake, add to manufacturing complexity without extra benefit). We also note that some common fractals suffer from large operating inefficiencies (for example, non-branching fractals such as Hilbert and Peano curves will experience large power losses in their electrodes). That said, the purpose of our study is to demonstrate the potential of fractals and a more comprehensive future study might identify better contenders.

**Fig 3:**
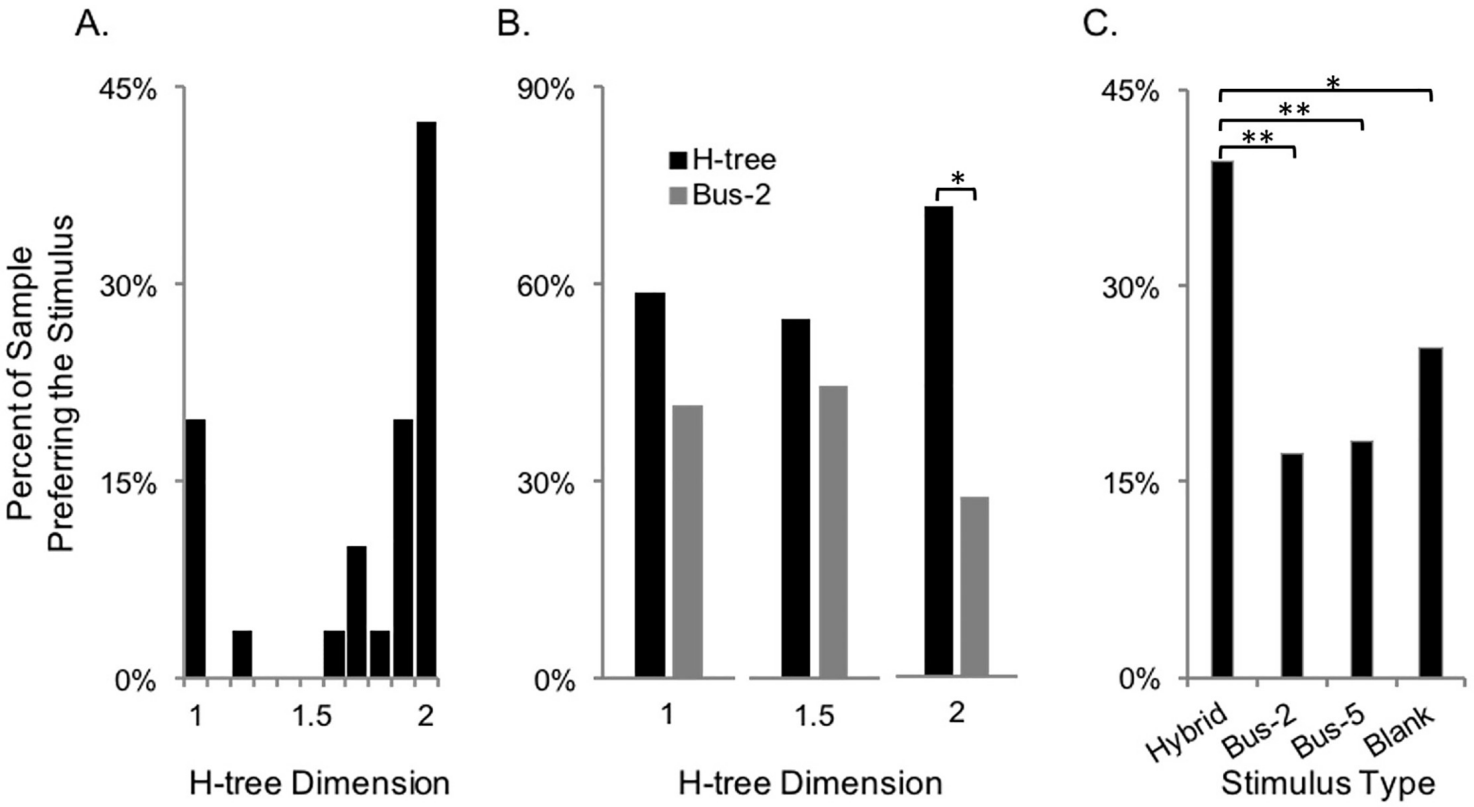
Visual preferences. A) Preferences for H-trees with different *D* values, B) Preferences for a 2-bus bar pattern compared to H-trees with *D* = 1, 1.5 and 2, C) Preferences for the hybrid, 2-bar and 5-bar patterns, and blank images. Significance levels with p < 0.05 are indicated by * and those with p < 0.01 by **.

Fig 1 (top row) shows the result of varying the H-tree pattern’s fractal dimension, *D*, which sets the rate at which the H shrinks with each repetition. Details of our H-tree generation technique are provided in the Methods. To study the aesthetic appeal of the H-tree, we first asked participants to indicate which H-tree they found most aesthetically appealing out of a set of 11 different H-trees with *D* values ranging from 1 to 2 in steps of 0.1. Details of our experimental design and analysis are presented in the Methods. A chi-square goodness of fit test revealed a significant number of individuals preferred H-trees with high *D* (χ^2^ = [10, *N* = 34] = 65.00, *p* < 0.001, Fig 3A). (Because no participants reported preference for the categories 1.1, 1.3, 1.4, or 1.5, we also performed a chi square test on the 7 options that participants chose and found similar results (χ^2^ = [6, *N* = 34] = 32.82, *p* < .001).) A smaller contingent expressed preference for low *D* H-trees, consistent with the conclusions of previous work in which cluster analysis revealed two groups of individuals who either preferred high or low levels of *D*, particularly if the patterns exhibited symmetry [23].

To address the question of how aesthetic H-tree fractals are relative to the bus-bar, our second study used head-to-head comparison questions to probe participants’ preference for H-tree and bus-bar patterns (Fig 1). Here we observed that preferences for the H-tree and bus-bar were not significantly different when the H-tree scaled at *D* = 1 (χ^2^ = [1, *N* = 29] = 0.86, *p* = 0.35, Fig 3B) or *D* = 1.5 (χ^2^ = [1, *N* = 29] = 0.29, *p* = 0.59, Fig 3B), consistent with the results from our first study where such fractal patterns were not preferred by the majority of individuals. However, significantly more individuals preferred the *D* = 2 H-tree to the bus-bar (χ^2^ = [1, *N* = 29] = 5.83, *p* = 0.02) (Fig 3B). These two studies provide evidence that the *D* = 2 H-tree is aesthetically preferable for the majority of people. Considering that aesthetics is a major concern for many consumers [4], this superior appearance can be expected to influence their likelihood of installation.

### Electrical properties of electrode patterns

**Fig 4:**
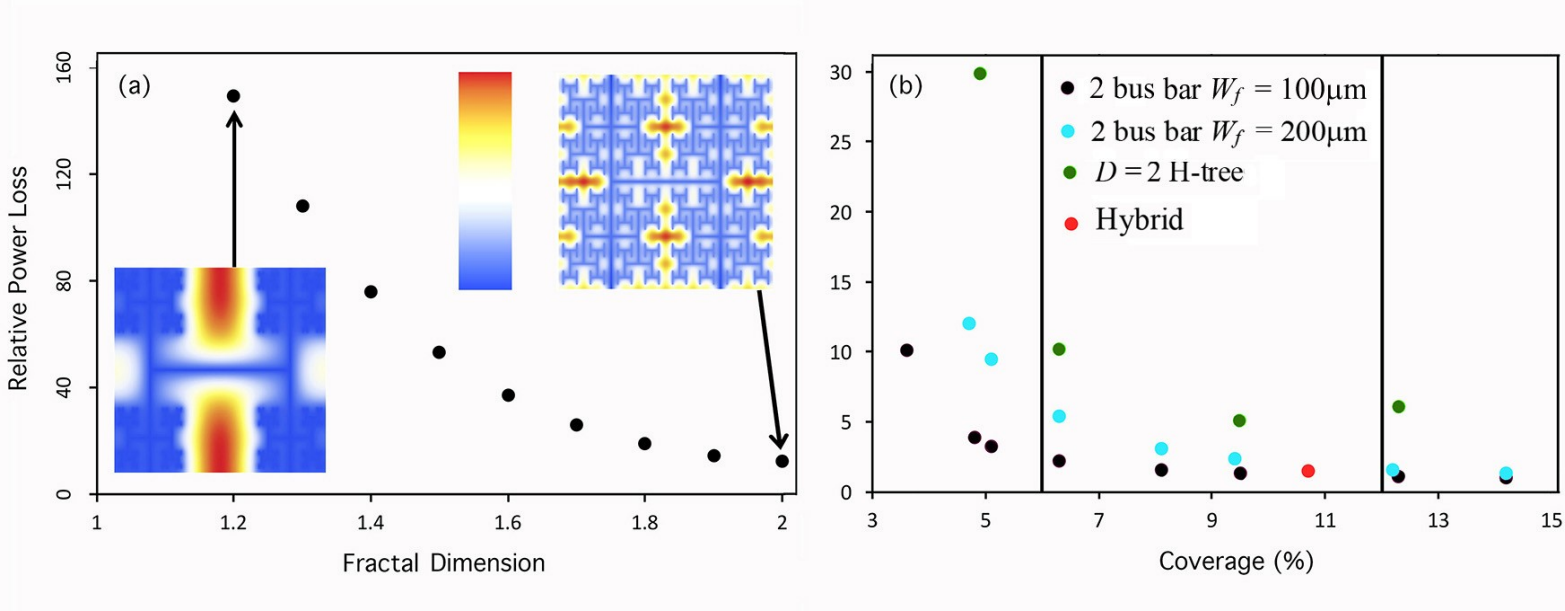
Power losses. (a) Power losses of H-trees with *D* values in the range 1.2–2.0. Each H-tree has *m* = 4 magnification layers with the same *W*_*h*_ dependency outlined in Fig 3. The voltage maps for the *D* = 1.2 and *D* = 2.0 H-trees are shown bottom-left and top-right respectively. Each pixel in these maps has a color dependent on its voltage. The darkest blue pixels in each map represent 0V. The scale is linear for both maps, but the maximum voltage values represented by red differ: 2.998V for the *D* = 1.2 H-tree and 0.216V for the *D* = 2.0 H-tree. All power loss values are again unitless and calculated relative to the bus-bar from Fig 3. (b) Relative power loss plotted against electrode coverage for *D* = 2 H-trees with increasing *m* = 3–6 from left to right (green), and 2 bus-bar designs featuring 2 vertical bars and finger widths of *W*_*f*_ = 100μm (black) and 200μm (blue). The varying coverages of these 2 bus-bar designs were achieved by using different numbers and therefore spacings *G*_*f*_ of the fingers: *G*_*f*_ varied from 800μm (left) to 6400μm (right). *W*_*b*_ = 1mm and *G*_*b*_ = 3.3cm for both bus-bars. The hybrid pattern (red) is an integration of the *D* = 2, *m* = 3 H-tree with the 2 bus-bar pattern shown in Fig 1 (bottom right), and is quantified by *W*_*b*_ = 800μm, *G*_*b*_ = 5cm, *W*_*f*_ = 100μm and *G*_*f*_ = 2mm. The vertical lines represent the approximate limits for the range of typical coverages for mass-produced bus-bar electrodes.

To address the question of how these patterns performed electrically, we used Modified Nodal Analysis [42] to simulate the electrical performance of a 10cm^2^ solar cell featuring a 50μm thick aluminum electrode positioned above a 1μm thick emitter layer of n-doped monocrystalline silicon (Fig 2 inset). Details of the Modified Nodal Analysis are presented in the Methods. The two coarse-scale vertical lines of the H-trees are extended to the pattern edges such that they act as bars for the electrical analysis. Fig 2 demonstrates that the total power loss associated with a *D* = 2, *m* = 6 H-tree electrode exceeds that of a bus-bar electrode with matching coverage of the emitter surface. For both cells, the power losses at the semiconductor-metal junction are negligible. The power losses in the metal dominate because of the large resistances generated by the narrow electrode widths required to maximize light transmission into the underlying cell. The H-tree suffers a greater power loss in the metal compared to the bus-bar because of the larger distances traversed by the current before exiting the electrode. The H-tree also suffers more power loss in the semiconductor because the average distance from a semiconductor pixel to the nearest electrode edge exceeds that for a bus bar. As expected, Fig 4A shows that reducing the H-tree’s *D* value exacerbates this problem due to the increasingly larger gaps in the electrode design. The voltage maps in Fig 4A reveal the associated bigger build-up of voltage in the larger gaps of the *D* = 1.2 design compared to the *D* = 2 design.

Fig 4B tunes these power losses by adjusting the bus-bar and H-tree parameters. The increased coverage of the bus-bars results from introducing more fingers. This reduces the average distance traversed by the current through the semiconductor emitter layer and produces the observed decrease in power loss. The increased coverage of the H-tree results from introducing more pattern iterations and this generates the observed ‘U’ shaped behavior as follows. For patterns featuring *m* = 4 levels or more, the dominant power losses occur in the metal and this power loss increases with *m* due to the larger typical distance traversed by the current before exiting the electrode. For patterns featuring fewer than *m* = 4 levels, the simultaneous decrease in metal resistance and increase in semiconductor resistance leads to semiconductor-dominated power losses. This increases as *m* decreases due to the appearance of larger gaps in the electrode design. To reduce the larger power losses of the fractal design to be close to those of the bus-bars while still lying within the coverage areas typical of mass-produced cells, we created a “hybrid” electrode design by incorporating bus-bars *and* fingers into a *D* = 2, *m* = 3 H-tree to produce a hybrid pattern (see Fig 1, bottom right). Crucially, this design modification produced power losses equivalent to those of the mass-produced bus-bar cells (Fig 4B). In particular, increasing *m* beyond three repetitions increases the power losses in the electrode and generate power losses in excess of the bus-bars.

### Aesthetic properties of the hybrid H-tree/Bus-bar pattern

Having developed a new hybrid H-tree/bus bar pattern for its electrical qualities, we turned to the question of whether the hybrid pattern inherits the H-tree’s superior fractal aesthetics. The aesthetic appeal of the hybrid pattern was compared to that of a 2-bar bus-bar (Fig 1, bottom, left), 5-bar bus-bar (Fig 1, bottom, middle), and solid (blank) image. In this image selection, we only considered the one hybrid pattern (*D* = 2, *m* = 3) that matched the power loss performance of the bus-bars. The fractal H-trees were similarly excluded because, although their power losses are close to those of standard bus-bars (see Fig 4B), we assume that the solar industry will only embrace new designs if their power losses are comparable to existing designs. We asked participants to indicate which pattern they preferred most. Details of our experimental design and analysis are presented in the Methods. The number of individuals expressing preference for the hybrid pattern (*n* = 83), 2-bar bus bar (*n* = 36), 5-bar bus-bar (*n* = 38) and blank image (*n* = 53) were not equally distributed (χ^2^ = [1, *N* = 210] = 26.91, *p* < 0.001) (Fig 3C). Pairwise comparisons revealed that the hybrid was chosen significantly more often than the 5-bar (χ^2^ = [1, *N* = 121] = 16.74, *p* < 0.001), the 2-bar (χ^2^ = [1, *N* = 119] = 18.56, *p* < .001) and the blank image (χ^2^ = [1, *N* = 136] = 6.62, *p* = 0.01), confirming the hybrid’s superior aesthetics. (Pairwise comparisons consider only the individuals who preferred one of the two categories being compared, so *N* varies from test to test.)

## General discussion

Although a significant body of psychology research highlights the positive visual qualities of fractals [23–36], only two studies have considered ‘exact’ fractals similar to those employed here [23, 25]. The remaining studies considered ‘statistical’ fractals, which introduce randomness into their construction. This previous research indicated that larger *D* fractals were perceived to have higher complexity than lower *D* fractals and that aesthetic preference was determined by this complexity [31]. Preference for the statistical fractals was found to peak for *D* = 1.3–1.5, which corresponds to the most prevalent *D* values in nature’s scenery [23–36]. Due to the absence of randomness, exact fractals are inherently less complex than their statistical counterparts, and this induces a tolerance for higher *D* values in the observer [23]. Thus, the peak preference shifts to higher *D* for exact fractals, particularly for fractals featuring symmetries that further simplify the pattern. The peak in preference for our *D* = 2 H-trees is consistent with this behavior. The absence of multi-scale repetition within the bus-bar and blank images explains their lower aesthetic quality–their complexities fall below the optimal amount set by exposure to nature’s fractals. The aesthetics results in our studies are therefore consistent with the large body of research examining the positive responses of viewing statistical fractals.

Given the larger amount of research on statistical fractals, it is reasonable to ask why not consider statistical rather than exact H-trees for solar applications? The answer is twofold: 1) Fortuitously, the aesthetic peak of *D* = 2 for the H-trees also corresponds to the peak in their electrical performance (Fig 4A), making them the ideal fractal for optimizing aesthetic and electrical functions. Given statistical fractals are randomized versions of exact fractals, their electrical performance will also peak at *D* = 2, which does not coincide with their aesthetic peak at *D* = 1.3–1.5. This mismatch between aesthetic and electrical performances makes them less ideal than their exact counterparts because optimizing the balance between their two functions will be harder to achieve for the statistical fractals, 2) The randomness in their patterning presents a significant construction challenge for the cost-driven solar industry.

Among the simpler fractal patterns that would be readily manufacturable, the H-tree is an optimal fractal for hybridizing with bus-bars, which results in an electrode with power losses that approximate to those of pure bus-bars (Fig 4B). We hope that our approach of iteratively performing experimental aesthetic studies and electrical simulations to validate new electrode patterns will be adopted for a broad range of applications, especially engineering and design. The hybrid design is aesthetically preferred over both bus-bars and blank images (which corresponds to cells with electrodes positioned on the back surface). When the hybrid electrode is incorporated into 10cm solar cells, its finest fractal features are separated by 3mm and can be resolved by observers with 20/20 vision from 10m (corresponding to roof-mounted panels viewed from an adjacent side walk). This observation range can be extended by adapting the patterns to create one large fractal spanning the entire panel. This larger fractal extends the observation range to approximately 100m. Although our participants viewed the stimuli with an upright orientation, panels will most likely be slanted at an angle. However, previous studies suggest that tilting doesn’t reduce pattern recognition abilities [48]. This study provides significant contributions to the field in terms of characterization of the aesthetics of the electrodes. Additional factors related to aesthetics, such as matching the panel’s color to the surroundings (e.g., a roof), further impact preference [49] have been noted at the individual consumer level, while social acceptance [50] and cost [51] are significant considerations as well. Future studies should therefore be conducted within the context of the roof-top environment (for example, a limitation of the current study was that participants were not informed that the patterns were being considered for solar panel application) to test the relative importance of various factors that influence solar adoption to build on prior work [4]. Finally, we note that glass louvers [5] could be patterned with fractals. However, incorporating louvers reduces light transmission and increases manufacturing costs, making fractal electrodes the optimal approach.
